# Direct impact of psoriasis on gingival crevicular fluid levels of VEGF-A in periodontitis patients: a mediation analysis

**DOI:** 10.3389/fimmu.2024.1477587

**Published:** 2024-10-24

**Authors:** Constanza Jiménez, Javier Fernández, Camila Rodríguez, Juan Felipe Mancilla, Elizabeth Pellegrini, Marcela Hernández, Fernando Valenzuela, Alejandra Fernández

**Affiliations:** ^1^ Facultad de Odontología, Laboratorio de Odontología Traslacional, Dermoral, Universidad Andres Bello, Santiago, Chile; ^2^ Departamento de Dermatología, Centro Internacional de Estudios Clínicos (CIEC) Probity Medical Research, Santiago, Chile; ^3^ Facultad de Odontología, Laboratorio de Biología Periodontal, Universidad de Chile, Santiago, Chile; ^4^ Departamento de Dermatología, Clínica Universidad de los Andes,Universidad de los Andes, Santiago, Chile; ^5^ Departamento de Dermatología, Facultad de Medicina, Universidad de Los Andes, Santiago, Chile

**Keywords:** psoriasis, periodontitis, vascular endothelial growth factor A, angiogenesis, gingival crevicular fluid, mediation analysis

## Abstract

**Introduction:**

Emerging evidence suggests that psoriasis and periodontitis are linked via systemic inflammation. However, the role of angiogenesis as an additional connecting mechanism between these diseases remains unclear.

**Methods:**

This case control study explored the effect of psoriasis on the gingival crevicular fluid (GCF) levels of vascular endothelial growth factor A (VEGF-A) in patients with different stages of periodontitis. Thirty-one patients with psoriasis (P) and thirty healthy controls (C) underwent physical and intraoral evaluations, with diagnoses confirmed by dermatologists and periodontists. GCF VEGF-A was measured using a multiplex-bead immunoassay. Statistical analyses included Fisher exact tests, Student’s T-tests, linear regression models, and mediation analyses.

**Results:**

Psoriasis patients had significantly lower GCF VEGF-A levels compared to controls (*p*=0.008). Psoriasis was negatively associated with GCF VEGF-A (*p*=0.006), while severe periodontitis was positively associated with GCF VEGF-A levels, regardless of tobacco use (*p*=0.027). Further analyses revealed that severe periodontitis significantly increased GCF VEGF-A levels only in the C group (*p*=0.038), but not in psoriasis patients (*p*>0.610). Mediation analyses confirmed a significant direct and total effect of psoriasis on GCF VEGF-A (*p*>0.002), with no significant indirect effect through periodontitis (*p*=0.699).

**Discussion:**

Psoriasis and severe periodontitis are associated with GCF levels of VEGF-A in opposite and independent ways. In subjects with psoriasis, the impact of the dermatosis is direct with no mediation from periodontitis.

## 1 Introduction

Psoriasis is a chronic inflammatory skin disease that predominantly affects individuals with genetic susceptibility (1). Although the precise mechanisms contributing to its development remain unclear, it has been well-established that the disease results from dysregulated interactions between the adaptive and innate immune responses (2). Similarly, periodontitis is a highly prevalent chronic inflammatory disease that targets and progressively destroys the supporting structures of the teeth (3). Its development and progression are strongly connected with polymicrobial dysbiosis of the oral microbiome and dysregulated immune responses from the host (4).

Over the past decade, both psoriasis and periodontitis have been recognized as significant public health concerns due to their high prevalence and epidemiological association with several chronic, inflammation-driven disorders, including diabetes (5, 6), metabolic syndrome (7, 8), and cardiovascular disease (7, 9, 10). These associations increase morbidity and mortality among affected individuals (11). Psoriasis and periodontitis also share overlapping comorbidities, common risk factors (e.g. tobacco use), and parallel pathogenic features, including shared immunological and genetic mechanisms (12, 13). For instance, the activation of the IL-17/Th1/Th2 signaling pathways has been reported to play a crucial role in the development of both psoriatic skin lesions (14) and periodontal bone loss (15, 16). Additionally, a recent bioinformatics study revealed further commonalities, such as the upregulation of the NF-kB, chemokine, cytokine-cytokine receptor, and AGE-RAGE signaling pathways (17). These findings underscore the overlapping nature of psoriasis and periodontitis, suggesting a profound and intricate interrelationship between both diseases.

Emerging evidence over the last decade recognizes a potentially positive and bidirectional relationship between psoriasis and periodontitis (15, 17–20). Specifically, individuals with periodontitis have an increased risk of developing psoriasis compared to periodontally healthy controls (21, 22), while those with psoriasis are twice as likely to suffer from periodontitis compared to non-psoriatic individuals (23). Given the independent association of these diseases with elevated levels of circulating proinflammatory cytokines and cells (24–26), it is theorized that their correlation could be driven by low-grade systemic inflammation (23, 26). While periodontitis contributes to bacteremia and systemic inflammation, which may trigger or intensify immune-mediated diseases such as psoriasis (27), the mechanisms by which psoriasis affects periodontitis remain unclear, as does the role of angiogenesis in this relationship.

Angiogenesis is the biological event by which new blood vessels are formed from pre-existing vascular networks (28). While the process is rare in adults, it may be aberrantly induced by chronic inflammation (29). In addition, angiogenesis is known to exacerbate and prolong chronic inflammation, thereby contributing to the pathogenesis of inflammatory-driven diseases such as psoriasis and periodontitis (29, 30). Vascular endothelial growth factor A (VEGF-A) is a critical protein in angiogenesis, known to induce vascular dilatation and increased vascular permeability, significantly contributing to inflammatory processes (31). While the local and systemic roles of VEGF-A in psoriasis and periodontitis have been previously explored separately (32–36), its specific function in the interrelationship between these two diseases remains unclear. We hypothesize that psoriasis influences the gingival crevicular fluid (GCF) levels of VEGF-A, and that is mediated by periodontitis. Accordingly, this study aimed to explore the effect of psoriasis on the gingival crevicular fluid (GCF) levels of VEGF-A in patients with different stages of periodontitis.

## 2 Materials and methods

### 2.1 Study design

This case control study was approved by the Scientific and Bioethics Committee of the Faculty of Dentistry at Andres Bello University, Santiago, Chile (approval no. PROPRGFO_002023_42). Informed consent was obtained from all participants before their enrollment. The research conformed to national and international ethical standards in compliance with the Helsinki Declaration (37). The manuscript was prepared with attention to the “Strengthening the Reporting of Observational Studies in Epidemiology (STROBE)” guidelines to ensure comprehensive and transparent reporting (38).

### 2.2 Sample size determination

The sample size for this study was calculated based on previously reported serum concentrations of VEGF-A in healthy subjects and patients with psoriasis (39). The reported VEGF-A serum levels were 459 ± 49 pg/mL for psoriasis patients and 228 ± 18 pg/mL for controls, yielding an effect size of 6.25. For a more conservative estimate, we considered an effect size of 0.8, with a significance level of α = 0.05 and a power of 0.8. These calculations indicated a minimum requirement of 26 individuals per group.

### 2.3 Participants

Thirty-one psoriasis patients (P) without additional systemic conditions and thirty systemically healthy controls (C) were purposefully recruited for this study. Enrollment took place from May to June 2023 at the International Center for Clinical Studies (CIEC) facilities and the Dental Clinic of the Faculty of Dentistry at Andres Bello University (UNAB) in Santiago, Chile. Eligible participants met the following inclusion criteria: (a) adults of legally consenting age (18 years or older) with (b) at least 12 teeth, excluding third molars. Exclusion criteria included individuals with (a) the presence of dermatological disorders other than psoriasis (e.g. lupus, rosacea, lichen, etc.), (b) patients with systemic immunoinflammatory conditions (e.g., diabetes, lupus, and rheumatoid arthritis), (c) current pregnancy, (d) treatment with antibiotics, anti-inflammatory, or immunomodulatory drugs within the last three months, (e) chemo- or radiotherapy within the past year, and (f) any treatment for psoriasis or periodontitis within the last three months.

### 2.4 Physical and intraoral evaluations

Participants underwent physical and intraoral evaluations, as previously described (18, 40, 41). Sociodemographic data and medical/dental histories were documented using predefined Excel charts. A trained dermatologist diagnosed psoriasis considering personal and familial medical records and the manifestation of hallmark clinical features such as well-defined, erythematous, and scaly skin plaques, along with symptoms like pruritus, burning sensation, and pain. Psoriasis severity was determined using the Psoriasis Area and Severity Index (PASI), as previously described (18, 40, 41). A qualified periodontist diagnosed periodontitis following full-mouth periodontal examinations. Key periodontal parameters were recorded at six sites per tooth using a manual periodontal probe (UNC-15, Hu-Friedy, IL, USA), including (a) bleeding on probing (BOP), (b) periodontal probing depth (PD), and (c) clinical attachment loss (CAL). Periodontitis was diagnosed based on the joint case definition proposed by the American Academy of Periodontology (AAP) and the Centers for Disease Control and Prevention (CDC) of the United States of America (42). After sample collection, patients diagnosed with periodontitis were immediately referred to the Periodontology Teaching Clinic at the Faculty of Dentistry of Andres Bello University for further treatment.

### 2.5 Gingival crevicular fluid sampling and determinations

A trained periodontist consistently collected GCF samples from the deepest site in each quadrant, as previously outlined (18, 40, 41). To avoid saliva contamination, the chosen sites were meticulously air-dried and isolated using sterile cotton rolls. Periodontal paper strips (PerioPaper, Oraflow, Plainview, NY, USA) were gently inserted into the gingival sulcus or pocket until resistance was felt. After 30 seconds, the strips were carefully removed and placed into sterile 2 mL tubes (Eppendorf, Eppendorf AG, Hamburg, Germany) for transport and storage at -20^a^C. Samples were then processed at the Periodontal Biology Laboratory, located at the Faculty of Dentistry of the University of Chile, Santiago, Chile. An experienced team of technicians performed all sample determinations, as previously described (18, 40, 41).

For the analysis, pooled samples from each participant were prepared by adding forty microliters of protein buffer to each tube. These dilutions were then incubated at 4°C for thirty minutes, and then centrifuged at 12.000 g for 5 minutes at the same temperature. This process was repeated twice to ensure efficient protein isolation. All samples were then diluted in a 1:50 ratio using the provided buffer. Following this, the samples were analyzed using a human multiplex bead immunoassay (Human Magnetic Luminex Assay, R&D Systems, Minneapolis, MN, USA), according to the manufacturer’s guidelines. VEGF-A levels in the GCF were quantified using a digital Magpix platform (Magpix, Millipore, MO, USA), utilizing de MILLPLEX AnalystR software version 5.1 (MILLPLEX AnalystR, Viagene Tech, MA, USA).

### 2.6 Statistical analyses

Statistical analyses were conducted using the STATA software version 14 (StataCorp. LLC, TX, USA). All tests were set at a significance level of 0.05 with a 95% confidence interval. Data distribution and homoscedasticity of variances were evaluated using Shapiro-Wilk and Levene’s tests, respectively. Since the data were normally distributed, quantitative variables were expressed as means and standard deviations (SD), whereas qualitative variables were expressed as frequencies and percentages. Inferential intergroup analyses were undertaken using Fisher’s exact test for categorical variables and Student’s t-test for continuous variables. Additional univariate and multivariate linear regression models were employed to further explore the influence of psoriasis, periodontitis, and other covariates (e.g., age, sex, smoking habit, etc.) on GCF levels of VEGF-A. Finally, mediation analyses were performed to further explore the mechanisms underlying the association between psoriasis and GCF VEGF-A, considering periodontitis as a mediating variable.

## 3 Results

This study included sixty-one individuals, divided into two groups: thirty-one patients with psoriasis (P) and thirty systemically healthy controls (C). Detailed information on participants’ demographics, smoking habits, and periodontal clinical parameters is presented in **Table 1**. Significant intergroup differences were observed in terms of CAL and smoking habits. Overall, the P group presented greater periodontal destruction, as indicated by mean CAL (*p*=0.044), despite having similar periodontal diagnoses and fewer smokers than the C group (*p*=0.390 and *p*=0.015, respectively).

**Table 1:** Comparative analysis of demographics, smoking habits, periodontal clinical parameters, and periodontal health status in psoriasis and control patients.

Parameters	C (n=30)	P (n=31)	*p*
Age (mean[SD])	36.97(13.66)	45.06(18.54)	0.224
Female (n, %)	15(50.00)	18(58.06)	0.422
Male (n, %)	15(50.00)	13(41.94)	
Smokers (n, %)	12(40.00)	9(29.03)	**0.015**
PD (mm, mean[SD])	2.18(0.52)	2.44(0.58)	0.058
CAL (mm, mean[SD])	1.90(1.07)	1.93(1.45)	**0.044**
BOP (%, mean[SD])	26.98(21.31)	22.06(13.00)	0.284
No Periodontitis (n, %)	7(23.33)	11(35.48)	0.390
Mild periodontitis (n, %)	7(23.33)	3(9.68)	
Moderate periodontitis (n, %)	8(26.67)	10(32.26)	
Severe periodontitis (n, %)	8(26.67)	7(22.58)	

C, control group; P, psoriasis group; PD, probing depth; CAL, clinical attachment level; BOP, bleeding on probing; SD, standard deviation; n, number; mm, millimeter; p, p-value, **bold:** p<0.05 (95% confidence interval).

**Figure 1:**
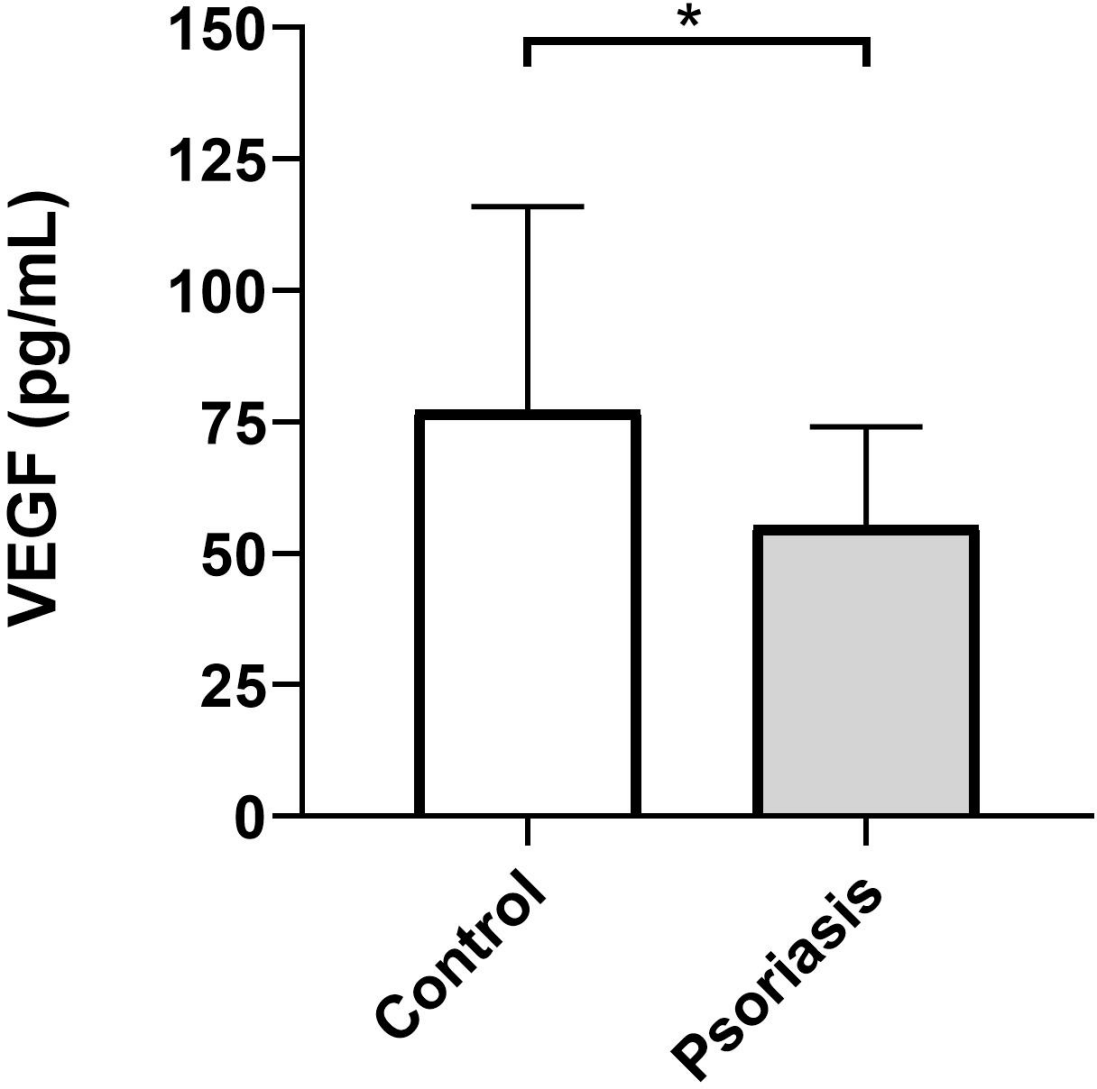
Differential VEGF levels in the gingival crevicular fluid of psoriasis individuals and healthy controls. Column bar graph plotting mean and standard deviations. Two-sample Student’s t-test, two-tailed; *, p=0.008.

The levels of VEGF-A detected in the GCF are illustrated in **Figure 1**. Psoriasis patients presented significantly lower levels of GCF VEGF-A compared to healthy controls (*p*=0.008). Specifically, patients in the P group had a mean ± SD of crevicular VEGF-A of 55.35 ± 18.75 pg/mL, whereas participants in the C group showed a higher mean ± SD of 77.26 ± 38.70 pg/mL.

Subsequently, a univariate linear regression analysis was performed to assess the association between psoriasis diagnosis and GCF levels of VEGF-A. The results revealed a negative association between psoriasis and GCF levels of the growth factor (*p*=0.006, **Table 2**). Likewise, a separate multivariate linear regression analysis was conducted to investigate the association between periodontitis severity and GCF levels of VEGF-A. These results showed that only severe forms of periodontitis were positively associated with the GCF levels of the growth factor (*p*=0.027, **Table 2**). To ensure the robustness of these findings, a comprehensive multivariate linear regression model was employed. This model accounted for tobacco use, a known risk factor for both the development and progression of psoriasis and periodontitis. Results indicated that smoking did not significantly alter the observed associations (*p*=0.241, **Table 2**), confirming that the opposing associations of psoriasis and severe periodontitis with GCF levels of VEGF-A were true regardless of tobacco use.

**Table 2:** Impact of psoriasis, periodontitis, and tobacco use on the GCF levels of VEGF.

Variables	VEGF-A	
	Coef. ± SE	*p*
Psoriasis	-21.91 ± 7.75	**0.006** ^1^
Cons.	77.26 ± 5.22	**0.000** ^1^
F(1, 59)	8.00	
Prob>F	0.006	
Adjusted R^2^	0.105	
Mild periodontitis	3.30 ± 12.33	0.790 ^1^
Moderate periodontitis	11.38 ± 12.33	0.280 ^1^
Severe periodontitis	24.83 ± 10.92	**0.027** ^1^
Cons.	56.12 ± 7.37	**0.000** ^1^
F(3, 57)	1.90	
Prob>F	0.139	
Adjusted R2	0.043	
Psoriasis ^2^	-23.40 ± 7.77	**0.004** ^2^
Mild periodontitis ^2^	-5.79 ± 11.97	0.631 ^2^
Moderate periodontitis	9.54 ± 9.80	0.335 ^2^
Severe periodontitis	21.56 ± 10.32	**0.041** ^2^
Tobacco use	-9.58 ± 8.08	0.241 ^2^
Cons.	74.15 ± 9.20	**0.000** ^2^
F(5, 55)	3.22	
Prob>F	0.013	
Adjusted R2	0.156	

^1^Univariate linear regression models; ^2^Multivariate linear regression model; **bold:** p<0.05 (95% confidence interval). Coef., coefficient; SE, standard error; p, p-value. Number of observations = 61.

**Figure 2:**
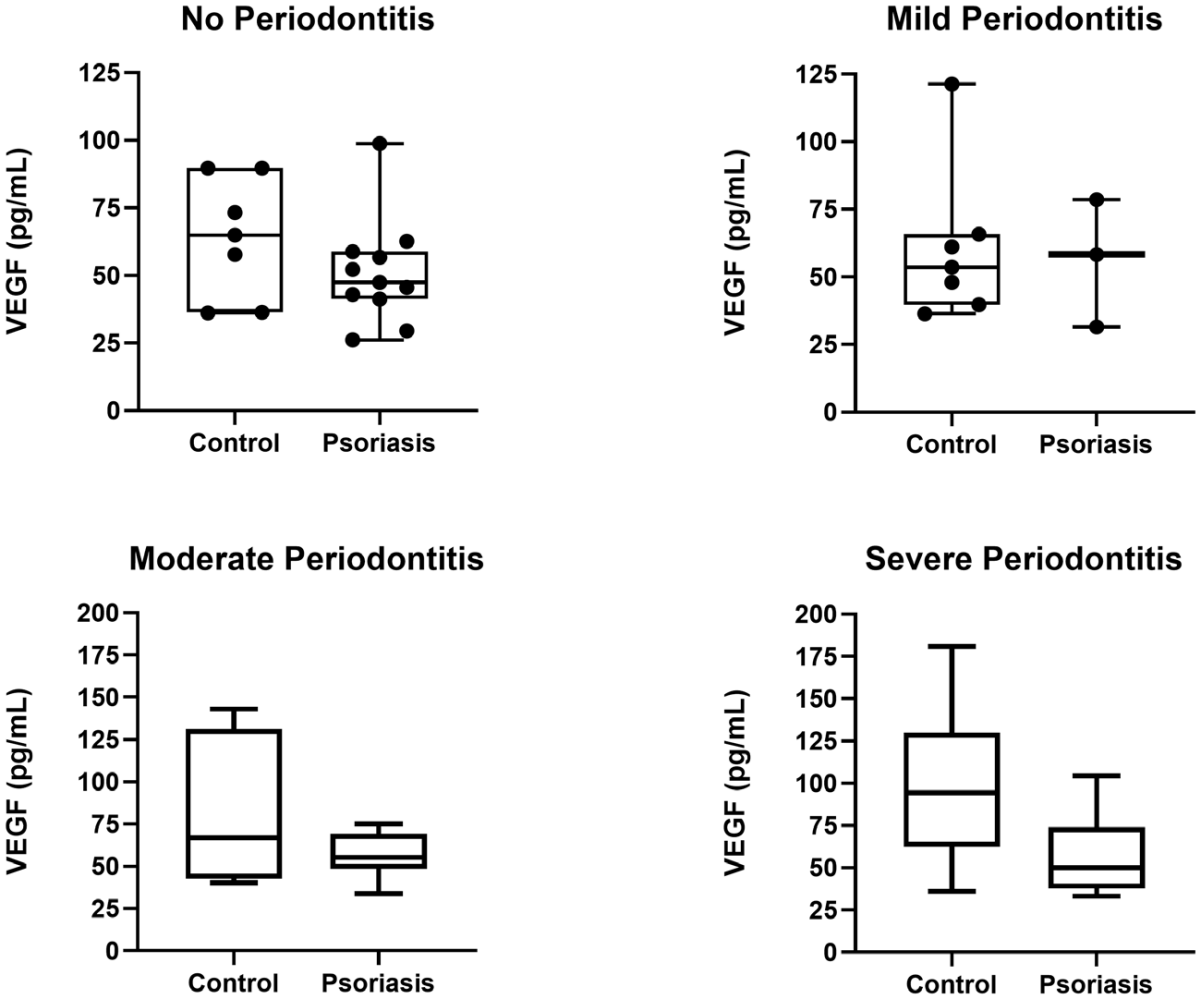
VEGF-A levels in psoriasis patients and healthy controls stratified by periodontal diagnosis.

Consequently, we explored how psoriasis and severe periodontitis interacted based on the GCF levels of VEGF-A among groups. This was done using multiple linear regression models (**Table 3**). In the psoriasis group, no significant associations between severe periodontitis and the GCF levels of VEGF-A were found (*p*>0.610). Conversely, in the control group, severe periodontitis was statistically associated with an increase in the crevicular levels of VEGF-A (*p*=0.038, **Table 3**, **Figure 2**). To ensure the strength of these findings, we performed an additional multivariate linear regression model accounting for covariates such as age and smoking habit (**Table 3**). The results indicated that these covariates did not significantly influence the observed associations (*p*≥0.236). Therefore, these results suggest that severe periodontitis is associated with increased GCF levels of VEGF only in the absence of psoriasis, whereas the association loses its significance in individuals with dermatosis.

**Table 3:** Interaction of periodontitis severity on the GCF levels of VEGF-A according to dermatological diagnosis.

Variable	Periodontitis Severity	VEGF-A	
		Coef. ± SE	*P*
Control	Mild	-3.15 + 15.83	0.843
	Moderate	17.23 + 15.33	0.266
	Severe	35.28 + 15.33	**0.025**
Psoriasis	Mild	-7.87 + 20.44	0.702
	Moderate	-7.48 + 14.59	0.610
	Severe	-3.98 + 15.83	0.803
	Cons.	64.00 ± 11.19	0.000
	F(7, 53)	2.41	
	Prob>F	0.032	
	Adjusted R2	0.141	
Control	Mild	-3.11 + 15.93	0.846
	Moderate	20.65 + 15.90	0.200
	Severe	35.87 + 16.82	**0.038**
Psoriasis	Mild	-11.21 + 21.73	0.608
	Moderate	-9.67 + 15.50	0.536
	Severe	-3.18 + 21.13	0.881
Smoking habit	—	-10.43 + 8.70	0.236
Age	—	0.02 + 0.36	0.960
	Cons.	66.41 ± 16.34	0.000
	F(9, 51)	2.02	
	Prob>F	0.055	
	Adjusted R2	0.133	

Multivariate linear regression model, Coef., coefficient; SE, standard error; p, p-value; **bold:** p<0.05 (95% confidence interval). Number of observations = 61.

**Figure 3:**
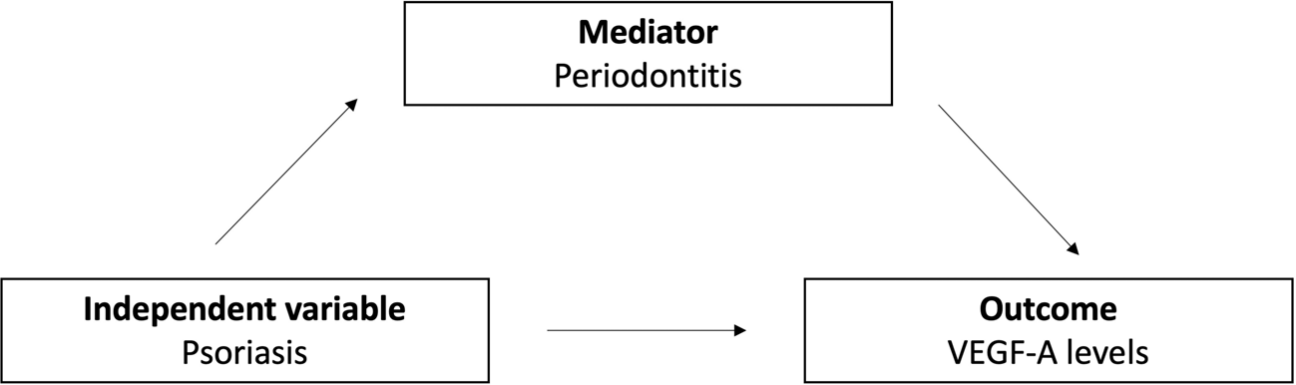
Model representation of the mediation analysis.

Finally, we conducted a mediation analysis to explore whether the association between psoriasis and GCF levels of VEGF-A in the P group could be mediated by the presence of periodontitis (**Table 4**). This analysis aimed to determine if the effect of psoriasis on GCF VEGF-A was direct or indirect, with periodontitis acting as an intermediary factor or mediator (**Figure 3**). The results showed a significant direct effect of psoriasis on GCF levels of VEGF-A (*p*=0.004), accounting for an estimated 96.8% of the total effect with the confidence interval excluding zero (-40.25 to -7.52). In contrast, the indirect effect of psoriasis on VEGF-A through periodontitis was minimal (3.2%) and not significant (*p*=0.699), indicating that periodontitis does not mediate this relationship. The total effect of psoriasis on GCF levels of VEGF-A was also significant (*p*=0.002), emphasizing the direct impact of psoriasis on the crevicular levels of the growth factor in patients with dermatosis. Therefore, while psoriasis significantly affects the GCF levels of VEGF-A, the mediating role of periodontitis in psoriasis patients appears negligible. It’s important to note that this mediation analysis was adjusted for potential confounders including age, sex, and smoking consumption.

**Table 4:** Direct, Indirect, and Total Effects of Psoriasis and Periodontitis on the GCF levels of VEGF-A.

Effect	Coef.	CI-L	CI-U	*p*
Direct (psoriasis → VEGF-A levels)	-23.884	-40.249	-7.519	**0.004**
Indirect (psoriasis → periodontitis → VEGF-A levels)	-0.789	-4.792	3.214	0.699
Total effect	-24.673	-40.490	-8.856	**0.002**

VEGF-A, Vascular endothelial growth factor A; Coef., coefficient; CI-L, lower bound of the Confidence Interval; CI-U, Upper bound of the Confidence Interval; p, p-value; **bold:** p<0.05 (95% confidence interval).

## 4 Discussion

Evidence shows that psoriasis and periodontitis are associated through systemic inflammation (15, 17–20), yet the role of angiogenesis in this relationship remains unexplored. GCF is a serum transudate secreted into the oral cavity from the dentogingival sulcus (43). Its production is interstitial, resulting from the osmotic gradient flowing from the periodontal plexus to the gingival crevice (44, 45). Containing analytes from both systemic circulation and periodontal tissues, GCF serves as a promising and non-invasive sampling method for investigating both diseases. Distinct cytokine profiles have been identified in GCF from individuals with and without inflammatory diseases like diabetes (46), rheumatoid arthritis (47, 48), psoriasis (40, 41, 49), rosacea (50), and atopic dermatitis (51). Thereby, in this study, we further explored the influence of psoriasis on GCF composition by measuring VEGF-A levels in subjects with and without psoriasis, and with different stages of periodontitis.

In psoriasis, VEGF-A plays a crucial role in new blood vessel formation, promoting inflammation and disease severity (52–54). Research supports this by showing higher VEGF-A expression and increased micro vessel density in the papillary dermis of psoriatic skin lesions compared to healthy skin (55). *In vitro* studies also show that VEGF-A expression is associated with increased VEGF receptors (VEGFR) 1 and 2 in endothelial cells and keratinocytes (56), highlighting the role of the VEGF-A/VEGFR axis in the abnormal cell proliferation and inflammation characteristic of psoriasis.

Recent studies indicate that the serum levels of VEGF-A are elevated in psoriasis patients compared to healthy controls (57, 58), suggesting that VEGF-A can translocate from skin lesions to the systemic circulation. Consequently, we anticipated to find higher GCF VEGF-A levels in psoriasis patients compared to controls, expecting a positive association between psoriasis diagnosis and GCF VEGF-A in psoriasis patients. However, our results showed the exact opposite: GCF VEGF-A levels were significantly lower in psoriasis patients compared to controls, and psoriasis was negatively associated with GCF VEGF-A, regardless of tobacco consumption. Furthermore, our mediation analysis demonstrated that psoriasis has a substantial and direct effect on GCF VEGF-A levels in diseased subjects (accounting for 96.8% of the total effect), whereas mediation through periodontitis was negligible (accounting for only 3.2% of the total effect). This findings prompt us to revise plausible mechanisms by which psoriasis may be influencing GCF VEGF-A in diseased individuals, including the prospective role of psoriasis-related VEGFRs.

Soluble VEGFR-1 (sVEGFR-1) is an antagonist of VEGF-A that regulates angiogenesis in both physiological and pathological conditions (59–61). It is primarily produced by non-endothelial cells such as monocytes, macrophages, epithelial cells, dendritic cells, and vascular muscle cells (59–61). By binding to VEGF-A, sVEGFR-1 inhibits angiogenesis by reducing the amount of free VEGF-A available to activate membrane-bound VEGFR-1 and -2 receptors on endothelial cells (59). In psoriasis, sVEGFR-1 production is increased in both skin lesions (55, 62, 63) and serum of diseased individuals (53, 64), suggesting its translocation from the skin to the systemic circulation. Moreover, sVEGFR-1 expression correlates with psoriasis activity (53, 65), indicating that it helps to regulate psoriasis pathogenesis by limiting angiogenesis. In this study, GCF VEGF-A levels were significantly lower in psoriasis patients compared to controls. These findings suggest that psoriasis-derived sVEGFR-1 in the systemic circulation prevents circulating VEGF-A produced in psoriasis from reaching the periodontal tissues and the GCF in diseased subjects.

Another mechanism by which psoriasis may reduce GCF VEGF-A levels in diseased individuals compared to controls could involve the endothelial barrier of periodontal tissues. Previous studies have shown that activation of the VEGF-A/VEGFR-1 pathway in endothelial cells of the periodontium increases the permeability, vasodilatation and apoptosis of the periodontal plexus (36). This enhances the osmotic gradient responsible for producing GCF, resulting in higher total volumes and flow rates of this biofluid at the dentogingival junction (66). In our study, patients with psoriasis exhibited lower GCF VEGF-A levels compared to controls. We believe this may occur because psoriasis-derived VEGF-A activates membrane-bound VEGFRs -1 and -2 in endothelial cells of the periodontal plexus, increasing the total volume and flow rate of GCF, further diluting VEGF-A in diseased individuals.

Periodontitis is characterized by angiogenic alterations, including the formation of loop-like blood vessels and increased vascular permeability. These changes facilitate the influx of proinflammatory cells, cytokines, and growth factors, exacerbating periodontal inflammation and destruction (36, 67). Research shows that VEGF-A is significantly overexpressed in periodontal tissues from periodontitis patients compared to those with gingivitis and healthy periodontium, and this overexpression is strongly associated with both the presence and severity of periodontitis (67–71).

Our study supports these observations, showing a significant and positive association between severe periodontitis and increased levels of GCF VEGF-A, regardless of tobacco consumption. However, when we stratified the data by groups and periodontitis severity, this association remained significant only in the control group, losing its importance in psoriasis patients. These findings suggest that psoriasis may be interfering with the production and/or release of periodontitis-derived VEGF-A in periodontal tissues, weakening the association between severe forms of the disease and GCF VEGF-A levels in individuals with psoriasis.

Finally, our findings revealed that patients with psoriasis experience greater periodontal destruction (measured by mean CAL) compared to controls. This was observed despite both groups presenting similar periodontal diagnoses and levels of clinical inflammation (measured by BOP). Notably. the frequency of smokers was significantly lower in the psoriasis group compared to the control group. This was unexpected since tobacco consumption is a known risk factor for periodontitis and its progression (72). Consequently, we would have anticipated less periodontal destruction in the psoriasis group, which had fewer smokers compared to controls. These findings align well with prior investigations indicating that psoriasis patients exhibit increased alveolar bone loss compared to non-psoriatic controls (73, 74).

*In vitro* studies have demonstrated that fibroblasts, osteoblasts, and osteoclasts express membrane-bound VEGFRs (36, 71). Activation of these receptors by VEGF-A plays a crucial role in periodontal destruction by promoting collagen degradation (71), osteoclast proliferation (75), and alveolar bone resorption (76). Given these insights, we propose that psoriasis-induced VEGF-A translocate from the systemic circulation to periodontal tissues, where it binds to VEGFRs located in the aforementioned cells. This additional influx of VEGF-A, beyond what is locally produced in periodontitis, further exacerbates periodontal destruction in psoriasis patients. As psoriasis-derived VEGF-A interacts and binds with these receptors, its concentration within the osmotic gradient of the periodontal tissues would decrease, leading to lower levels of VEGF-A in the resultant GCF. This could be an additional explanation for the lower GCF VEGF-A levels observed in psoriasis patients compared to controls in this study.

### 4.1 Limitations

Findings from this study must be interpreted with caution due to design-related biases and limitations. As is common in observational studies, this research cannot establish a causal relationship between psoriasis and GCF VEGF-A. Additionally, it is not possible to prove temporal sequencing between the variables, and there may be unaccounted covariates that may have influenced the observed associations. Although we accounted for smoking status, we used a general measure of tobacco exposure rather than a detailed quantification of lifetime exposure (e.g. pack years). This may result in a more generalized assessment of smoking’s impact on our findings. Moreover, the sample size may limit the generalizability of our findings, impeding the immediate escalation of results to larger and more diverse populations. Therefore, more studies will be needed to further enhance the robustness and applicability of our results, as well as more research to fully understand the complex relationship between psoriasis, periodontitis, and angiogenesis.

## 5 Conclusions

In conclusion, both psoriasis and severe periodontitis are significantly associated with GCF VEGF-A in opposite and independent ways. Psoriasis lowers GCF VEGF-A levels, whereas severe periodontitis increases them. In patients with psoriasis, the positive association of severe periodontitis with GCF VEGF-A is completely mitigated, whereas results from the mediation analysis indicate that psoriasis diagnosis accounts for 96.8% of the total effects on GCF VEGF-A, with no significant mediation by periodontitis.

## Data Availability

The raw data supporting the conclusions of this article will be made available by the authors, without undue reservation.
